# Validity of the 2021 Traumatic Encephalopathy Syndrome Criteria for CTE pathology

**DOI:** 10.1002/alz70857_096744

**Published:** 2025-12-24

**Authors:** Jesse Mez, Brigid Dwyer, Michael L. Alosco, Belinda Yew, Alexandra Pritchett, Natalia Bernal Fernández, Amelia J Hicks, Bobak Abdolmohammadi, Shruti Durape, Madeline Uretsky, Megan H. Ryder, Farwa Faheem, Sophia Novak, Brett Martin, Joseph N. Palmisano, Christopher J. Nowinski, Yorghos Tripodis, Kristen Dams‐O'Connor, Lee E Goldstein, Douglas I Katz, Robert C. Cantu, Neil W Kowall, Robert A. Stern, Victor E. Alvarez, Bertrand Russell Huber, John F. Crary, Thor D. Stein, Daniel H. Daneshvar, Ann C. McKee

**Affiliations:** ^1^ Boston University Chobanian & Avedisian School of Medicine, Boston, MA, USA; ^2^ Icahn School of Medicine at Mount Sinai, New York, NY, USA; ^3^ Boston University Alzheimer's Disease Research Center, Boston University Chobanian & Avedisian School of Medicine, Boston, MA, USA; ^4^ Boston University Chronic Traumatic Encephalopathy Center, Boston, MA, USA; ^5^ Boston University Chronic Traumatic Encephalopathy Center, Boston University Chobanian & Avedisian School of Medicine, Boston, MA, USA; ^6^ Boston University School of Public Health, Boston, MA, USA; ^7^ Concussion Legacy Foundation, Boston, MA, USA; ^8^ Emerson Hospital, Concord, MA, USA; ^9^ Boston VA Medical Center, Boston, MA, USA; ^10^ VA Boston Healthcare, Jamaica Plain, MA, USA; ^11^ Harvard Medical School, Boston, MA, USA

## Abstract

**Background:**

**V**alidity of the 2021 NINDS Traumatic Encephalopathy Syndrome (TES) criteria, proposed to diagnose chronic traumatic encephalopathy (CTE) in life, has not been assessed.

**Methods:**

Brain donors were selected across 6 brain banks (15+ donors each), 9 repetitive head impact (RHI)/traumatic brain injury (TBI) groups (15+ donors each): college or professional American football; less than college football; college or professional contact sports, not football; less than college contact sports, not football; military combat, no contact sports; military combat and contact sports, concussion with loss of consciousness, no RHI; moderate to severe TBI, no RHI; no RHI/TBI; and 5 age groups (25+ donors each): 20‐34; 35‐49; 50‐64; 65‐79; 80+. Blinded to clinical information, neuropathologists applied NINDS/NIBIB CTE neuropathological criteria and staging (I‐IV). Blinded to neuropathological information, clinicians interviewed informants and reviewed medical records, and an expert panel adjudicated TES diagnoses, including provisional levels of CTE certainty (suggestive/possible/probable). Clinical and neuropathological diagnoses were *a priori* dichotomized for primary and age‐stratified analyses: TES with possible/probable CTE vs. no TES/TES with suggestive CTE; CTE stages II‐IV vs. no CTE/stage I CTE.

**Results:**

**Among** 193 brain donors [men: 153 (79.3%), mean age: 66.4 (SD:22.0), white race: 158 (81.9%)], 57 (29.5%) donors met clinical criteria for TES with possible/probable CTE and 42 (21.8%) donors met neuropathological criteria for CTE stages II‐IV. Using neuropathological diagnosis as the gold‐standard, TES criteria sensitivity, specificity, positive likelihood ratio (LR) and negative LR were overall: 0.79, 0.84, 4.9, 0.25; age 50: 0.93, 0.90, 9.6, 0.07; age <50: 0.42, 0.66, 1.22, 0.89. Twenty‐four donors who met clinical but not neuropathological criteria (false‐positives), had stage I CTE (5) or other pathologies including vascular disease (11), Alzheimer's disease (9), Lewy body disease (2), motor neuron disease (2) and limbic predominant age‐related TDP43 encephalopathy (1). Nine donors who met neuropathological but not clinical criteria (false‐negatives) had TES with suggestive CTE (3), another etiology fully explain the syndrome (4), insufficient RHI exposure (1) or inconclusive course (1).

**Conclusion:**

**The 2021** TES criteria were sensitive and specific for CTE pathology across a range of RHI/TBI exposures, particularly above age 50, raising optimism for use in clinical care.

